# Determination of Minimum Inhibitory Concentrations of Selected Antibiotics Against *Trueperella pyogenes* Originated from Bovine Clinical Endometritis

**DOI:** 10.3390/pathogens14050405

**Published:** 2025-04-24

**Authors:** Ottó Szenci, Ákos Jerzsele, Zoltán Somogyi, Ádám Kerek, Attila Répási, Lea Lénárt, László Makrai

**Affiliations:** 1 Department of Obstetrics and Food Animal Medicine Clinic, University of Veterinary Medicine Budapest, H-2225 Üllő, Hungary; lenart.lea@univet.hu; 2 Department of Pharmacology and Toxicology, István utca 2, University of Veterinary Medicine Budapest, H-1078 Budapest, Hungary; jerzsele.akos@univet.hu (Á.J.); somogyi.zoltan@univet.hu (Z.S.); kerek.adam@univet.hu (Á.K.); 3 National Laboratory of Infectious Animal Diseases, Antimicrobial Resistance, Veterinary Public Health and Food Chain Safety, University of Veterinary Medicine Budapest, István utca 2, H-1078 Budapest, Hungary; 4 Pataki Veterinary Ltd., Arany J. út 40, H-3944 Sárospatak, Hungary; repasia71@gmail.com; 5 Autovakcina Ltd., Szabadság sugárút 57, H-1171 Budapest, Hungary; info@autovakcina.hu

**Keywords:** cow, endometritis, *Trueperella pyogenes*, MIC values

## Abstract

Bacteriological examination of uterine secretions provides essential information for the prevalence of bovine uterine pathogens and their influence on fertility. The objective of the present study was to determine the uterine pathogens in cases of clinical endometritis in two Holstein-Friesian dairy farms between 21 and 27 days after calving and the minimum inhibitory concentration (MIC) of 14 antimicrobials for *Trueperella pyogenes* (*T. pyogenes*). Overall, the prevalence of *T. pyogenes* (Farms A and B) was 46.3% and 22% (*p* < 0.01), respectively. In contrast, Farm B had significantly more cases (*p* < 0.001) of *Escherichia coli*, but the distribution of uterine pathogens was similar. Regarding the prevalence of any bacteria, Farm B also had significantly more bacteria (*p* < 0.001) than Farm A. *T. pyogenes* isolates were highly susceptible to amoxicillin, amoxicillin/clavulanic acid, tylosin, and cephalosporins, such as ceftiofur, cefquinome, and cephalexin with MIC_90_ of ≤2 μg/mL. At the same time, MIC_90_ of tulathromycin, lincomycin, and florfenicol were between 4 and 8 μg/mL and of doxycycline, enrofloxacin, oxytetracycline, and gentamicin, were between 16 and 32 μg/mL, respectively. Meanwhile, sulfamethoxazole/trimethoprim showed the highest MIC_90_ (>32 μg/mL). In summary, *T. pyogenes* with high MIC_90_ against oxytetracycline, gentamicin, and sulfamethoxazole/trimethoprim were found, which calls attention to the prudent use of antibiotics.

## 1. Introduction

The primary task of a dairy farm is to reduce the period between two calvings to a minimum value, which can be significantly endangered by reproductive biological disorders resulting from uterine inflammations that develop after calving [1,2].

Three weeks after calving, clinical endometritis is characterized by the formation of mucous-purulent or purulent uterine discharge, while signs of systemic disease are not detectable [3,4,5]. Since most of the purulent vaginal discharge (PVD) is not associated with endometritis, it is advisable to separate these cases from clinical endometritis [6]. Clinical endometritis and PVD can affect approximately 6.5–35% of dairy cows 4–6 weeks after calving. According to Esslemont [7], the lowest and highest quartile prevalence values can vary between 3.7% and 26.9%. All this harms the cows’ chance of re-conception and the number of artificial inseminations, so they can cause significant economic wastage to individual dairy farms.

For a long time, it has been suggested to “perform regular bacteriologic examinations of uterine secretions and to test the sensitivity of the microorganisms that are present” on dairy farms because of the danger of developing antibiotic resistance. On the other hand, bacteriological examination of uterine secretions provides essential information for the incidence of microorganisms in cows with clinical endometritis and their influence on fertility [8]. Recently, the importance of regular testing of the sensitivity of the microorganisms increased with the appearance of “superbugs”, which are bacteria that have become resistant to more than one antibiotic, making them more challenging to treat effectively [9,10].

Aerobic and anaerobic uterine bacteria recovered by culture from the uterus can be classified as uterine pathogens, like *Trueperella* (*T.*) *pyogenes*, *Escherichia* (*E.*) *coli*, *Prevotella* spp., *Bacteroides* spp., *and Fusobacterium* (*F.*) spp.; potential pathogens, like *Bacillus licheniformis*, *Enterococcus faecalis, Mannheimia haemolytica, Pasteurella multocida, Peptostreptococcus spp., Staphylococcus aureus*; and non-hemolytic streptococci and opportunistic contaminants, like *Clostridium perfringens, Klebsiella pneumoniae, Micrococcus* spp., *Providencia stuartii, Proteus* spp., *Staphylococcus* spp. coagulase-negative, a-hemolytic streptococci, *Streptococcus acidominimus* and *Aspergillus* spp. [3,11,12]. The uterine pathogens used to be associated with uterine endometrial lesions, while the potential pathogens are not commonly associated with uterine lesions, and the opportunist contaminants are not associated with endometritis [3].

Several etiological agents have been associated with endometritis and PVD; of these, *T. pyogenes, E. coli,* and *F. necrophorum* are considered the most relevant uterine pathogens in dairy cows because their coordinated action results in inflammation and destruction of the endometrium [13,14,15]. These uterine pathogens can usually be isolated from swab samples [16,17,18].

Only some studies from Israel [19], the USA [20], Great Britain [21], China [22,23], and New Zealand [24] have specifically examined the minimum inhibitory concentration (MIC) of antimicrobials for *T. pyogenes* isolated from the bovine reproductive tract in cases of clinical endometritis. According to our present knowledge, there are no MIC data on isolates originating from endometritis in the Middle European region.

The primary aim of our study was to map the uterine pathogens that may be behind the disease processes in clinical endometritis in two Holstein-Friesian dairy farms. We also examined the antibiotic susceptibility of the *T. pyogenes* isolates by determining the MIC of 14 antibacterial agents that can be used in medical treatment in the field.

## 2. Materials and Methods

### 2.1. Farms and Cows

Bacteriological swab samples were collected in two commercial Holstein-Friesian dairy farms in Hungary, with around 1000 to 1500 lactating cows on each farm. Cows were housed indoors in free-stall barns with straw bedding. Before calving, cows in each farm were fed a prepartum total mixed ration (TMR) ad libitum, containing a dietary forage-to-concentrate ratio of 78:22 on a dry matter (DM) basis. After calving, cows were fed a postpartum TMR ad libitum with a 60:40 forage-to-concentrate ratio on a DM basis. Water was available ad libitum [25].

### 2.2. Sample Collection

Rectal palpation and uterine massage were commonly performed as part of the control of involution between days 21 and 27 to diagnose clinical endometritis at each farm. Clinical endometritis was supposed to occur when mucopurulent or purulent discharge appeared in the vulva after rectal manipulation of the uterus [3,4,5].

Uterine bacteriological swab samples from Farm A were collected from the uterine body of cows using uterus culture swabs (Ref: 17214/2950, Minitüb GmbH, Tiefenbach, Germany). The sterilized culture swab had an introduction pipette and a pre-perforated cap for sampling without risk of contamination. The vulva was first dry-cleaned with cellulose wipes, and after opening the vulvar lips with one of our hands, the uterus culture swab was inserted into the vagina and passed through the cervix into the uterus. The internal swab was then pushed through the rubber cap of the external sheath, and the swab was turned to bring it into contact with the uterine contents. The swab was then retracted into its sheath and gently pulled out of the uterus. After that, the swab was pushed through the rubber cap again, removed, and inserted into the transport medium (sterile transport swab, Sarstedt, Nümbrecht, Germany) and placed in a cooler bag.

Vulvar bacteriological samples from Farm B were collected after dry-cleaning the vulva with cellulose wipes, opening the vulvar lips with one of our hands using sterile transport swabs (Sarstedt), and placing them in a cooler bag. The samples were stored in a refrigerator at 4 °C until delivery to the laboratory. In all cases, the swab samples were delivered to the laboratory within 24 h.

Altogether, 108 samples were collected for bacteriological examinations from the uterus (Farm A: *n* = 67) and from the vulva (Farm B: *n* = 41).

### 2.3. Isolation and Identification of Bacterial Pathogens

Swab samples were inoculated onto two blood agar plates (one for aerobic and one for anaerobic culture) (Biolab Ltd., Budapest, Hungary), one *Campylobacter*-selective agar (MERCK Ltd., Budapest, Hungary), one MacConkey agar (Biolab Ltd., Budapest, Hungary), and one Sabouraud agar (Biolab Ltd., Budapest, Hungary). One of the blood agar plates was incubated in an anaerobic environment, the *Campylobacter* plate in microaerophilic, and other plates under aerobic conditions were supplemented with 10% CO_2_ at 37 °C for 72 h. Pure cultures were prepared from the bacterial colonies in the primary cultures. Pure cultures were identified at the genus level based on cultural, morphological, and biochemical features. Identification of bacterial isolates at the species level was carried out by using matrix-assisted laser desorption ionization—time of flight (MALDI-TOF) (Bruker Daltonik, Bremen, Germany).

### 2.4. MIC Determination 

The in vitro susceptibility tests of 36 *T. pyogenes* isolates (Farm A: *n =* 27, Farm B: *n =* 9) were performed at the Department of Pharmacology and Toxicology, University of Veterinary Medicine Budapest. All MICs were performed in cation-adjusted Mueller–Hinton broth (Mueller–Hinton Broth II, Merck KGaA, Darmstadt, Germany) and 5% (*v*/*v*) fetal bovine serum supplementation (Fetal Bovine Serum, Merck, Darmstadt, Germany) was used, following the Clinical and Laboratory Standards Institute (CLSI) specifications, so that our results are reproducible and internationally comparable with those of other laboratories [26]. For each isolate, the final concentration of bacteria in the broth dilution was 5 × 10^5^ colony-forming units per mL (CFU/mL). Broth dilutions were performed on 96-well microplates (96-well BRANDplates–F–pureGrade S, VWR International, Radnor, PA, USA). The 96-well microplate was used to prepare a two-based dilution series of the following antibacterial agents: amoxicillin, amoxicillin-clavulanic acid (2:1 ratio), cefalexin, ceftiofur, cefquinome, doxycycline, enrofloxacin, florfenicol, gentamicin, lincomycin, oxytetracycline, sulfamethoxazole-trimethoprim (1:19 ratio), tulathromycin, and tylosin. The concentration range of antibacterial agents was selected based on quality control principles and clinical limits [26,27].

### 2.5. Statistical Analysis

Data were analyzed using R3.5.2 [28]. Numeric variables were checked for normality with a Shapiro–Wilk test. Means, standard deviations, and percentages were calculated for comparisons.

Fischer’s exact test was used to compare the number of pathogens isolated at Farms A and B. Differences in MIC values for each antibacterial agent were analyzed using the Kruskal–Wallis rank-sum test with Farm as a factor. The probability of *p* ≤ 0.05 was deemed statistically significant.

## 3. Results

The prevalence of culture-positive cases, irrespective of bacterial species, was 88.1% (Farm A) and 97.6% (Farm B) between days 21 and 27, respectively (Table 1). Overall, the prevalence of *T. pyogenes* was 46.3% and 22%, and the difference between the two farms became significant (*p* < 0.01). In contrast, Farm B had significantly more cases (*p* < 0.001) of *E. coli*, but the distribution of uterine pathogens was similar between the two farms. Regarding the culture cases with any bacteria, Farm B also had significantly more bacteria (*p* < 0.001) than Farm A (Table 1).

The *T. pyogenes* strains originated from Farms A (*n =* 27) and B (*n =* 9), so the MIC values were evaluated first according to the farms. Since the two farms had no significant differences, the data obtained were evaluated in combination.

The frequency distributions of MIC values for *T. pyogenes*, including the MIC_50_ and MIC_90_, are presented in Table 2. In total, 3%, 3%, 28%, 31%, and 100% of the *T. pyogenes* isolates were resistant (based on the interpretive criteria from human-derived data) for tulathromycin, enrofloxacin, oxytetracycline, gentamicin, and trimethoprim/sulfamethoxazole, respectively.

As shown in Table 2, all *T. pyogenes* isolates were highly susceptible to amoxicillin, amoxicillin/clavulanic acid, tylosin, and cephalosporins, such as ceftiofur, cefquinome, and cephalexin with MIC_90_ at ≤2 μg/mL. At the same time, MIC_90_ of tulathromycin, lincomycin, and florfenicol were between 4 and 8 μg/mL. Relatively high MIC_90_ of 16 and 32 μg/mL were found for doxycycline, enrofloxacin, oxytetracycline, and gentamicin, respectively. Meanwhile, sulfamethoxazole/trimethoprim showed a very high MIC_90_ value (>32 MIC_90_).

## 4. Discussion

The present study’s primary aim was to determine the prevalence rate of uterine pathogens and any bacteria growth from clinical endometritis cases at two Hungarian dairy farms. The minimum inhibitory concentrations of selected antibiotics (*n =* 14) against *Trueperella pyogenes* were also performed. The prevalence rate of *T. pyogenes* was significantly higher (*p* < 0.01) in the examined Farm A (46.3%) than in Farm B (22%). In comparison, the prevalence rate of *E. coli* was significantly higher together with any bacteria growth (*p* < 0.001) in Farm B than in Farm A.

The prevalence rates of *T. pyogenes* and *E. coli* at six pasture-based dairy herds in New Zealand on day 21 after calving were 9% (range between herds, 4–17%) and 24% (17–37%), respectively, while any bacterial growth irrespective of species, was 79% (73–89%). The six herds had no significant differences regarding the prevalences of the two uterine pathogens [24]. In a recent study, Liu et al. [29] found a prevalence rate of 24.1% for *T. pyogenes* and 39.1% for *E. coli* in the case of endometritis on three dairy farms between days 21 and 34 after calving.

In the case of postpartum metritis, the prevalences of the two uterine pathogens (28.6% and 32.1%) were very close to Farm B (22% and 26.8%) [30]. The differences between the studies can be attributed to the different housing conditions (pasture-based vs. a confinement housing environment) and the different sampling methods (uterus vs. vagina), to which difference Dubuc et al. [6] also draw attention.

A broth microdilution susceptibility testing method for *T. pyogenes* of animal origin was recently developed [31] and included in the CLSI document VET06 [26]. Based on MIC distributions [31], breakpoints for the category “susceptible” have been proposed for penicillin, ampicillin, erythromycin, and trimethoprim-sulfamethoxazole [26].

Six studies determined the MIC values of 63 antibiotics against *T. pyogenes* originating from endometritis in dairy cows (Table A1 and Table A2). However, two different methods, broth microdilution [20,22,23,24] and agar dilution [19,21], were used, which must be considered when evaluating the results obtained [32].

Oxytetracycline was analyzed in each study, while enrofloxacin [19,21,22,24], or ceftiofur [21,22,24], clindamycin [20,22,24], and tetracycline [20,22,23], were examined in four and three different studies. Regarding the MIC_50_ values determined by the broth microdilution method, most of the various antibiotics had ≤2 μg/mL except amikacin (4 μg/mL), oxytetracycline (8 μg/mL, *n =* 2), ceftiofur (8 μg/mL), bacitracin zinc (≥32 μg/mL), streptomycin (≥64 μg/mL), sulfadiazine (≥128 μg/mL), and sulfamethoxydiazine (≥128 μg/mL) [20,22,23,24]. In contrast, when an agar dilution method was used to determine the MIC_50_ values, several antibiotics had ≤2 μg/mL except apramycin, cefotaxime, chloramphenicol, gentamicin, norfloxacin, and streptomycin, in each case with 3.12 μg/mL, while it was >10 μg/mL for sulfadiazine/trimethoprim or 16 and 50 μg/mL for oxytetracycline, respectively [19,21]. At the same time, the MIC_90_ values determined with the broth microdilution method showed wider varieties [20,22,23,24]. Sixteen antibiotics had <2 μg/mL, eight had between 4 and 8 μg/mL, while the remaining nineteen antibiotics had ≥16 μg/mL. When the MIC_90_ values in the different studies were determined with the agar dilution method, the MIC_90_ values were <2 μg/mL in most cases (*n =* 11). At the same time, they changed between 3.12 and 6.25 μg/mL in three cases and in the remaining 6 cases, between >10 and >100 μg/mL, respectively. Oxytetracycline had the highest value [19,21]. It is important to mention when *T. pyogenes* originated from the endometritis of pasture-based cattle [24], the MIC_90_ values of the nine different antibiotics were ≤1 μg/mL except for ceftiofur (2 μg/mL), which demonstrates a healthier environment compared with the confinement housing environment of other studies.

We determined the MIC_50_ values of 14 different antibiotics, and nine had ≤2 μg/mL values, while doxycycline, enrofloxacin, oxytetracycline, and gentamicin had 8 μg/mL values. In the case of sulfamethoxazole/trimethoprim, the MIC_50_ values were already >32 μg/mL.

In contrast, regarding MIC_90_ values, only six antibiotics remained in the ≤2 μg/mL, three in the 4–8 μg/mL, and five in the ≥16 μg/mL category, indicating that the proportions of resistant strains for the five antibiotics (doxycycline, enrofloxacin, oxytetracycline, gentamicin, and sulfamethoxazole-trimethoprim) were 19%, 42%, 42%, 42%, and 100%, respectively.

In a recent study, Liu et al. [29] examined 10 different antibiotics, such as azithromycin, cefazolin, ciprofloxacin, enrofloxacin, streptomycin, amoxicillin, gentamicin, kanamycin, ampicillin, and tetracycline, and *T. pyogenes* isolates originating from endometritis of dairy cows were defined as “susceptible”, “intermediate”, or “resistant” based on the MICs for each antimicrobial agent. The rate of the *T. pyogenes* strains becoming resistant against the different antibiotics in ascending order (ampicillin, azithromycin, kanamycin, tetracycline, amoxicillin) changed between 52.2% and 91.3%. More than 33% of isolated *T. pyogenes* strains were resistant to seven or more antibiotics, while more than 83% of isolated strains were resistant to three or more antibiotics.

Two studies used the broth microdilution method to determine the MIC values of different antimicrobial agents against *T. pyogenes* originating from metritis [33,34]. The rate of the *T. pyogenes* strains becoming resistant against the different antibiotics in ascending order (ceftiofur, florfenicol, penicillin, ampicillin, chloramphenicol) changed between >50% and 100%, respectively [33]. 29.2% of isolated *T. pyogenes* strains were resistant to six antibiotics, while 89.9% and 95.8% were resistant to three or two antibiotics. In contrast, Pohl et al. [34] determined the MIC_90_ values of 16 different antimicrobial agents against *T. pyogenes* originating from metritis had a better result because MIC_90_ was ≤2 μg/mL except for gentamicin (4 μg/mL) and tetracycline (64 μg/mL). Similar results (among 10 antimicrobial agents, only tetracycline had >4 μg/mL MIC_90_) were reported when examining *T. pyogenes* strains originating from mastitis using the same broth microdilution method [35], or when *T. pyogenes* strains originated from summer mastitis cases (among 16 antimicrobial agents (<0.5 μg/mL MIC_90_), only ofloxacin had 2 μg/mL MIC_90_ [36]. In a more recent study [37], 16 antimicrobial agents were examined against *T. pyogenes* originating from mastitis cases. The MIC values of each antibiotic agent in the majority of the cases were ≤2 μg/mL except for oxacillin, trimethoprim, kanamycin, and trimethoprim/sulphadoxazole when >10% of the MIC values (changed between 16.3% and 35.4% in ascending order) were between 4 and 8 μg/mL, respectively. Antibiotic treatment is still the primary method of treating bacterial infections in dairy practice, so bacterial resistance monitoring can provide a theoretical basis and guidance for clinical treatment medication. Continuous monitoring of recurrent infections and the difficulty of thoroughly eliminating infections on the same dairy farm is also an essential management task. The growing prevalence of multi-resistant *T. pyogenes* strains calls attention to the prudent use of antibiotics in dairy practice, even in humans, because a similar prevalence rate of resistant *T. pyogenes* strains was reported recently [38,39]. Recently used metagenomic sequencing techniques allow us to know the species composition and functional potential of the vaginal and uterine microbiota as well as the epidemiological characteristics and horizontal transfer mechanisms of antimicrobial resistance genes in this way and their roles in the development of uterine diseases, thereby helping to develop methods that do not require antibiotic treatments [40].

## 5. Conclusions

In conclusion, this study indicates that because antibiotic treatments are the primary method of treating clinical endometritis, continuous monitoring of bacterial resistance to select the appropriate antibiotics is essential for dairy management. The growing prevalence of multi-resistant *T. pyogenes* strains calls attention to the prudent use of antibiotics in dairy practice.

## Figures and Tables

**Table 1:** Cows with uterine pathogens and any other bacterial growth, irrespective of species (any bacteria), between days 21 and 27 after calving at two Hungarian dairy farms ^a^.

Pathogens	Farm A (*n* = 67)	Farm B (*n* = 41)
	*n* (%)	*n* (%)
*Trueperella pyogenes*	31 (46.3)	9 (22.0) **
*Escherichia coli*	1 (1.5)	11 (26.8) ***
*Prevotella* spp.	1 (1.5)	0
Uterine pathogens totals	33 (49.3)	20 (48.8)
Any bacteria	26 (38.8)	40 (97.6) ***
Culture-positive	59 (88.1)	40 (97.6)
Culture-negative	8 (11.9)	1 (2.4)

** *p* < 0.01; *** *p* < 0.001; ^a^ Identification of bacterial isolates at the species level was carried out by using matrix-assisted laser desorption ionization—time of flight (MALDI-TOF).

**Table 2:** Frequency distribution (% of all isolates; *n =* 36) of MIC for a range of antimicrobials against *Trueperella pyogenes* isolated between 21 and 27 DIM from postpartum bovine reproductive tracks.

Antimicrobials	<0.03	0.06	0.125	0.25	0.5	1	2	4	8	16	32	>32	MIC_50_	MIC_90_
Amoxicillin	22	67	6	6	0	0	0	0	0	0	0	0	0.06	0.06
Amoxicillin/Clavulanic acid	8	67	17	6	0	0	3	0	0	0	0	0	0.06	0.125
Tylosin	0	69	6	14	0	0	3	8	0	0	0	0	<0.06	0.25
Ceftiofur	50	25	19	6	0	0	0	0	0	0	0	0	0.03	0.125
Cefquinome	0	3	14	33	28	8	6	0	3	6	0	0	0.25	2
Cephalexin	0	0	0	0	0	11	78	11	0	0	0	0	2	2
Tulathromycin	0	0	0	0	0	17	36	39	6	0	3	0	2	4
Lincomycin	0	0	6	25	36	6	11	3	6	6	0	3	0.5	8
Florfenicol	0	0	0	0	0	11	56	19	3	11	0	0	2	8
Doxycycline	0	0	8	0	0	0	0	25	47	19	0	0	8	16
Enrofloxacin	0	0	0	0	3	17	14	14	11	39	3	0	8	16
Oxytetracycline	0	0	0	8	0	3	0	3	44	14	28	0	8	32
Gentamicin	0	0	0	0	0	0	3	8	47	11	28	3	8	32
Sulfamethoxazole/Trimethoprim	0	0	0	0	0	0	0	0	0	0	6	94	>32	>32

MIC_50_ and MIC_90_ = MIC (μg/mL) that inhibited 50 and 90% of the isolates, respectively.

## Data Availability

Data are contained within the article.

## Abbreviations

The following abbreviations are used in this manuscript:

PVD	Purulent vaginal discharge
CFU	Colony-forming units
MALDI-TOF	Matrix-assisted laser desorption ionization -time of flight
TMR	Total mixed ration
DM	Dry matter
CLSI	Clinical and Laboratory Standards Institute
MIC	Minimum inhibitory concentration
*T.*	*Trueperella*
*E.*	*Escherichia*

## Appendix A

**Table A1:** MIC_50_ and MIC_90_ values for a range of antimicrobials against *Trueperella pyogenes* isolated from bovine endometritis cases using the broth microdilution method.

References	Antimicrobial Agent(s)	MIC_50_	MIC_90_
Trinh et al. [20] (*n* = 6)	Chlortetracycline	0.12	8
	Clindamycin	≤0.06	64
	Erythromycin	≤0.06	64
	Oxytetracycline	0.25	8
	Tetracycline	0.25	16
	Tylosin	≤0.06	64
Liu et al. [22] (*n* = 23)	Amikacin	4	≥64
	Amoxicillin	1	4
	Azithromycin	0.25	0.25
	Bacitracin zinc	≥32	≥32
	Cefazolin	1	16
	Ceftiofur	8	16
	Ciprofloxacin	2	2
	Clindamycin	0.25	32
	Doxycycline	0.5	16
	Enrofloxacin	0.25	1
	Erythromycin	0.125	2
	Florfenicol	1	4
	Gatifloxacin	0.5	0.5
	Gentamicin	0.5	8
	Ofloxacin	2	2
	Oxacillin	1	4
	Oxytetracycline	8	32
	Penicillin G	2	4
	Streptomycin	≥64	≥64
	Sulfadiazine	≥128	≥128
	Sulfamethoxydiazine	≥128	≥128
	Tetracycline	1	32
	Tilmicosin	0.25	0.25
de Boer et al. [24] (*n* = 9)	Ampicillin	0.06	0.06
	Ceftiofur	1	2
	Cephapirin	0.25	0.25
	Cefuroxime	0.06	0.12
	Clindamycin	0.06	0.12
	Cloxacillin	0.25	0.5
	Enrofloxacin	1	1
	Oxytetracycline	0.5	1
	Ticarcillin/ Clavulanic acid	0.06	0.06
Zhang et al. [23] (*n* = 5)	Chlortetracycline	1	16
	Doxycyclin	0.5	16
	Metacycline	0.5	8
	Oxytetracycline	8	32
	Tetracycline	1	32

**Table A2:** MIC_50_ and MIC_90_ values for a range of antimicrobials against *Trueperella pyogenes* isolated from bovine endometritis cases using the agar dilution method.

References	Antimicrobial Agent(s)	MIC_50_	MIC_90_
Cohen et al. [19] (*n* = 14)	Amoxycillin	0.05	0.10
	Apramycin	3.12	6.15
	Cefotaxime	3.12	6.25
	Cephalothin	0.10	0.10
	Chloramphenicol	3.12	12.50
	Enrofloxacin	0.39	1.56
	Gentamicin	3.12	3.12
	Lincomycin	0.025	0.10
	Norfloxacin	3.12	12.50
	Oxytetracycline	50	>100
	Penicillin G	0.05	0.10
	Streptomycin	3.12	50
	Sulfadiazine/ Trimethoprim	>10	>10
	Tylosin	0.05	0.10
Sheldon et al. [21] (*n* = 6)	Ceftiofur	<0.06	0.125
	Cefquinome	<0.05	0.125
	Cephapirin	<0.06	<0.06
	Cephapirin/ Mecillinam	<0.06	0.06
	Enrofloxacin	1	1
	Oxytetracycline	16	32
